# DZIP3 is a key factor to stratify IDH1 wild-type lower-grade gliomas

**DOI:** 10.18632/aging.103817

**Published:** 2020-11-21

**Authors:** Tingyu Liang, Xingang Zhou, Peiliang Li, Gan You, Fang Wang, Peng Wang, Enshan Feng

**Affiliations:** 1 Department of Neurosurgery, Beijing Ditan Hospital, Capital Medical University, Beijing 100015, China; 2 Department of Pathology, Beijing Ditan Hospital, Capital Medical University, Beijing 100015, China; 3 Department of Neurosurgery, Beijing Tiantan Hospital, Capital Medical University, Beijing 100050, China

**Keywords:** DZIP3, glioma, IDH1 wild-type, survival time

## Abstract

Background: Malignant glioma is the most common form of primary malignant brain cancer. Heterogeneity is the hallmark of glioma. DAZ-interacting zinc finger 3 (DZIP3), acts as an RNA-binding RING-type ubiquitin ligase; however, its function in glioma is yet unclear.

Results: The *DZIP3* expression was related to the World Health Organization (WHO) grade and isocitrate dehydrogenase 1(*IDH1*) status, as well as the clinical outcome. Malignant cases exhibit lower *DZIP3* expression. *DZIP3* was an independent predictive factor of good prognosis in all grade and lower grade gliomas (*p* < 0.0001). Gene enrichment analysis and immunohistochemistry indicated that DZIP3 affected the biological behavior of glioma through the angiogenesis pathway. Moreover, based on *DZIP3* expression, *IDH1* wild-type lower-grade gliomas could be divided into two groups with different survival time.

Conclusion: In conclusion, the loss of DZIP3 may be involved in the mechanism of angiogenesis in the invasive biological process of glioma. These findings laid an understanding of DZIP3-specific clinical features in glioma.

Methods: A total of 325 glioma patients from the Chinese Glioma Genome Atlas (CGGA) RNA-seq cohort comprised the training cohort, while 265 patients from the GSE 16011 array cohort formed the validation cohort. The mRNA expression of *DZIP3* and clinical characteristics was assessed. DZIP3 protein expression and microvessel density (MVD) were evaluated by immunohistochemistry (IHC).

## INTRODUCTION

Gliomas are the most common and deadliest malignant primary brain tumor in adults and accounts for approximately 80% of the primary malignant tumors in the central nervous system [1]. Based on the World Health Organization (WHO) classification criteria, diffuse glioma could be divided into grades II–IV. Lower-grade gliomas (LGGs), designated as astrocytoma, oligodendroglioma, and mixed oligoastrocytoma of grade II and III, account for approximately 43.2% of all gliomas diagnosed in adults [2, 3]. Glioblastoma (GBM) is the most malignant glioma, which refers to WHO grade IV glioma. Despite advances in comprehensive therapy, such as neurosurgical resection, adjuvant radiotherapy, and alkylating agent temozolomide chemotherapy, patients who suffer from gliomas have a short median survival time due to various reasons [4, 5]. The degree of genetic and phenotypic variations of the glioma cells form intratumoral heterogeneity, allows the most adaptive tumor clones to develop treatment resistance [6]. In order to overcome this issue, hundreds of molecular alterations have been identified in grade II, III, and IV gliomas [7]. Isocitrate dehydrogenase 1 (*IDH1*) mutation, which occurs early in gliomagenesis, especially for WHO grade II and III gliomas, is an acknowledged molecular alteration [8]. Mutation in *IDH1* is a stable marker indicating prognosis in both LGGs and GBM with the incidence of 75% and 12%, respectively [9, 10]. However, previous studies have established gene signatures to divide the *IDH1* mutation glioma patients with different clinical features into various groups [9, 11]. However, only a few studies have focused on the wild-type *IDH1* patients. Therefore, there is an urgent need to identify specific genes for novel diagnosis and therapeutic strategies for human gliomas.

DAZ interacting zinc finger 3 (DZIP3) is first identified as an RNA-binding RING-dependent ubiquitin ligase [12]. It is involved in various physiological processes, such as regulation of chemokine- or estradiol-induced gene expression and self-renewal [13]. Ito et al. indicated that *DZIP3* affects the developmental genes in mouse embryonic stem cells by reorganizing the 3D chromatin conformation, and knockdown of *DZIP3* could result in an expansion of the mouse embryonic stem cells [12]. However, to the best of our knowledge, no study has explored the correlation between *DZIP3* and gliomas.

In the present study, we recruited 590 glioma patients from CGGA RNA-seq (training set), and GSE 16011 array (validation set) sets to compare the mRNA expression level of *DZIP3* from different clinical and molecular glioma subtypes. Gene enrichment analysis and immunohistochemistry (IHC) confirmed that DZIP3 could block glioma progression by affecting angiogenesis. Finally, in lower grade wild-type *IDH1* patients, based on *DZIP3* expression, two groups with different outcomes were established. One group was regarded as *IDH1* mutation-like, and the other group was GBM-like.

## RESULTS

### *DZIP3* expression was downregulated in GBM and *IDH1* wild-type glioma

**Figure 1:**
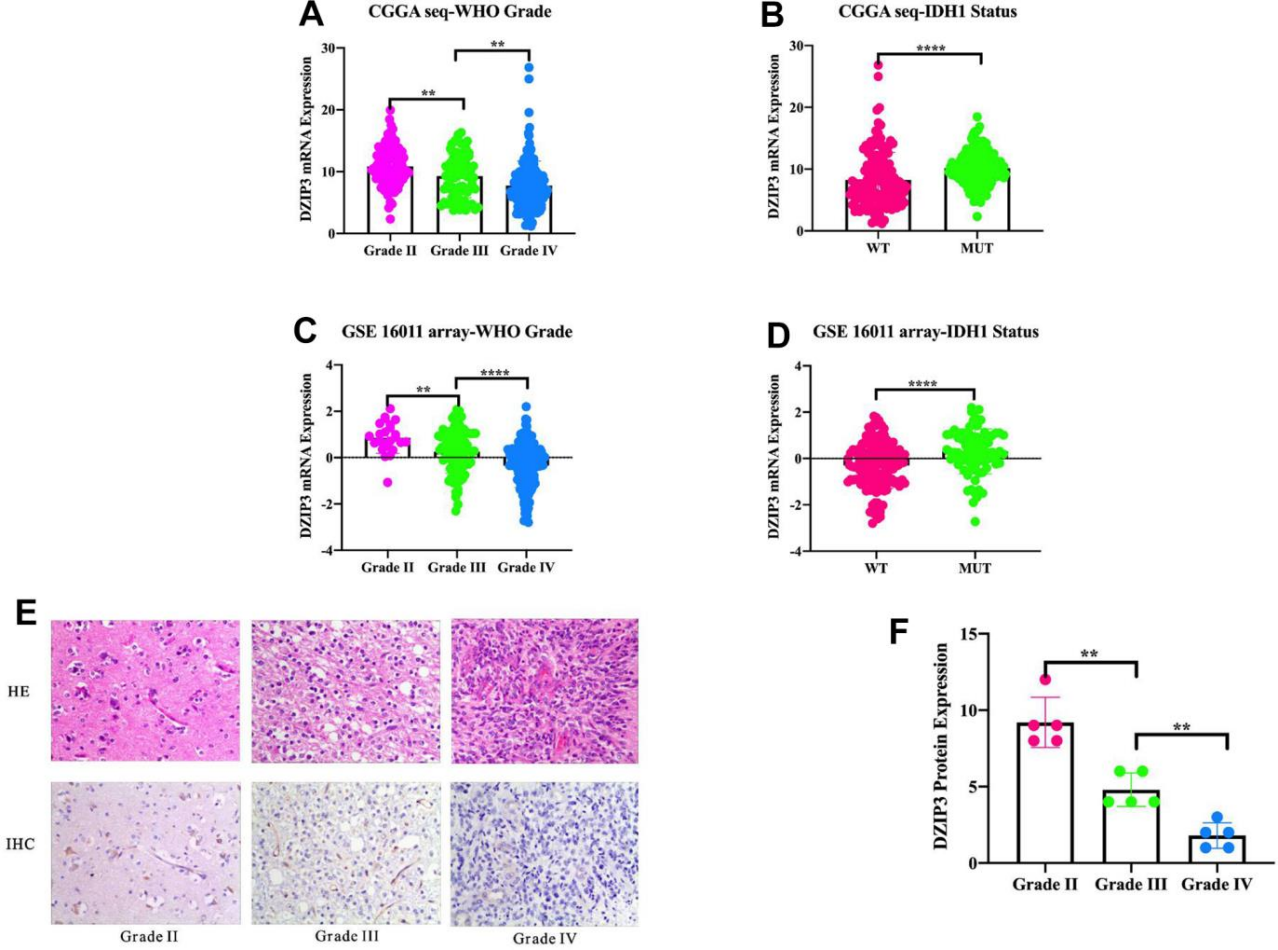
***DZIP3* mRNA expression pattern in CGGA RNA-seq and GSE 16011 array cohorts.** *DZIP3* is enriched in low-grade gliomas in CGGA RNA-seq and GSE 16011 array sets (**A**, **C**). *DZIP3* is enriched in *IDH1*-MUT gliomas in CGGA RNA-seq and GSE 16011 array sets (**B**, **D**). DZIP3 protein expression is negatively associated with WHO grade (**E**, **F**). **p<0.01 and ****p<0.0001.

*IDH1* status was increasingly recognized as the crucial genetic marker for glioma patients and had been included in the 2016 WHO classification of glioma. To get an overview of *DZIP3* status, we examined the expression pattern of *DZIP3* across WHO grade and *IDH1* status. The results demonstrated that *DZIP3* was significantly downregulated in GBM and *IDH1* wild-type groups (Figure 1A, 1B) in the CGGA RNA-seq set. Additionally, the results could be validated in 285 glioma patients from GSE 16011 array set (Figure 1C, 1D). To the best of our knowledge, GBM and *IDH1* wild-type were markers that indicated malignancy and short survival time in glioma. These results indicated that *DZIP3* worked as an anti-oncogene in gliomas. To verify the results above in protein level, we randomly selected 15 patients (5 for grade II, 5 for grade III, 5 for grade IV) to perform IHC to detect the relationship between tumor grade and DZIP3 protein expression and the results showed that DZIP3 protein expression was negatively associated with WHO grade (Figure 1E, 1F).

### DZIP3 was closely associated with angiogenesis function in glioma

**Figure 2:**
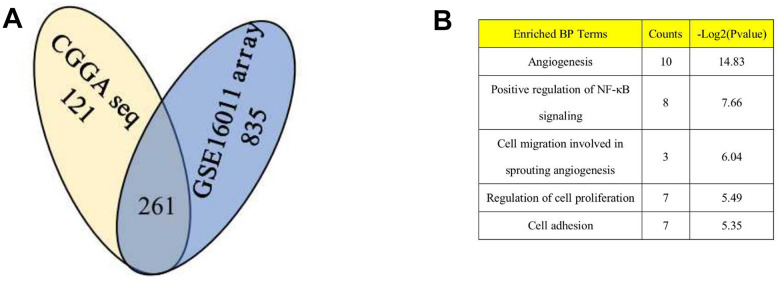
***DZIP3* is significantly associated to angiogenesis pathway in gliomas.** (**A**) 261 overlapping genes are negatively associated (Pearson |R| > 0.4) with *DZIP3* in CGGA RNA-seq and GSE 16011 array sets; (**B**) GO analysis of the 261 genes indicates that *DZIP3* is involved in angiogenesis, NF-KB and other GO function.

**Figure 3:**
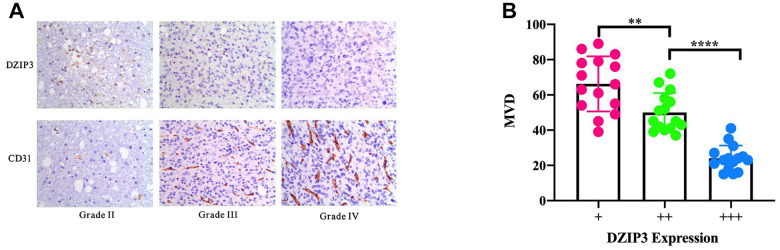
(**A**) IHC staining of DZIP3 and CD31 in different grades. (**B**) CD31-MVD is lower in samples with higher DZIP3 expression. **p<0.01 and ****p<0.0001.

Based on the aforementioned results, *DZIP3* affected the key biological functions to change the malignancy of glioma. A total of 261 genes were found to be significantly negatively related with the expression of *DZIP3* as assessed by Pearson’s correlation analysis (Pearson |R| > 0.4) in the CGGA RNA-seq and GSE 16011 array cohorts to explore the biological roles of *DZIP3* in gliomas (Figure 2A). Next, we performed GO analysis via DAVID Bioinformatics Resources 6.8 to clarify the biological functions of the related genes mentioned above. The results indicated that genes, negatively associated with *DZIP3*, were primarily involved in angiogenesis (Figure 2B). IHC was used to evaluate the correlation between DZIP3 expression and CD31 (a novel angiogenesis marker) [14]; DZIP3 mainly expressed on vascular endothelial cells and was negatively associated with CD31 at the protein level indicating that DZIP3 could affect the angiogenesis process of glioma (Figure 3A). Moreover, MVD of CD31 immunoreactivity was performed as described, we found that CD31-MVD was significantly lower in patients with higher DZIP3 expression (Figure 3B).

### High *DZIP3* mRNA expression confers improved survival time in all grade and lower grade glioma

**Figure 4:**
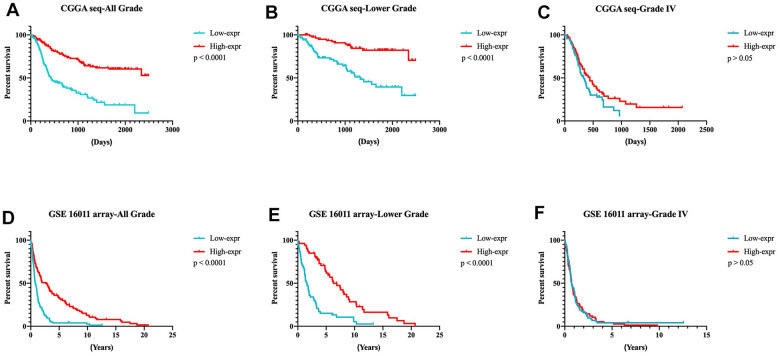
**Prognostic significance of DZIP3 in all-grade, lower-grade and GBM groups.** (**A**–**C**) CGGA RNA-seq cohort; (**D**–**F**) GSE 16011 array cohort.

In the CGGA RNA-seq set (training set), glioma patients were divided into two groups based on the median expression of *DZIP3*. Figure 4A, 4B showed that in both all grade and lower grade gliomas, patients in higher *DZIP3* expression groups possessed significantly longer survival time than lower expression counterparts (*p* < 0.0001). However, we cannot attain a similar tendency in the GBM group (Figure 4C, *p* > 0.05). Furthermore, the results above in the glioma patients from GSE 16011 array set were validated (Figure 4D–4F). Then, Cox regression analysis verified the independence of the clinical prognostic significance of *DZIP3* in glioma after adjusting age at diagnosis and WHO grade and *IDH1* status in both all grade and lower grade glioma in CGGA RNA-seq cohort (Table 1). These results indicated that DZIP3 plays a crucial biological function in gliomas, especially lower-grade glioma.

**Table 1:** Univariate and multivariate Cox analysis in CGGA RNA-seq cohort.

**Characteristic**	**Univariate analysis**		**Multivariate analysis**	
	**HR (95% CI)**	**P-value**	**HR (95% CI)**	**P-value**
**All grade**				
Age at diagnosis	1.039 (1.023-1.056)	< 0.0001	0.999 (0.983-1.015)	0.897
WHO grade	3.569 (2.751-4.629)	< 0.0001	2.791 (2.078-3.749)	< 0.0001
Gender	1.204 (0.837-1.731)	0.316		
IDH1 status	0.253 (0.172-0.370)	< 0.0001	0.574 (0.364-0.905)	0.017
DZIP3 expression	0.824 (0.780-0.870)	< 0.0001	0.927 (0.877-0.980)	0.007
**Lower grade**				
Age at diagnosis	1.047 (1.016-1.078)	0.003	1.017 (0.987-1.047)	0.274
WHO grade	5.921 (3.137-11.176)	< 0.0001	3.511 (1.734-7.110)	< 0.0001
Gender	1.027 (0.568-1.857)	0.93		
I DH1 status	0.274 (0.153-0.491)	< 0.0001	0.445 (0.227-0.873)	0.018
DZIP3 expression	0.765 (0.692-0.844)	< 0.0001	0.855 (0.776-0.943)	0.002

### *DZIP3* could further stratify *IDH1* wild-type lower-grade glioma

**Figure 5:**
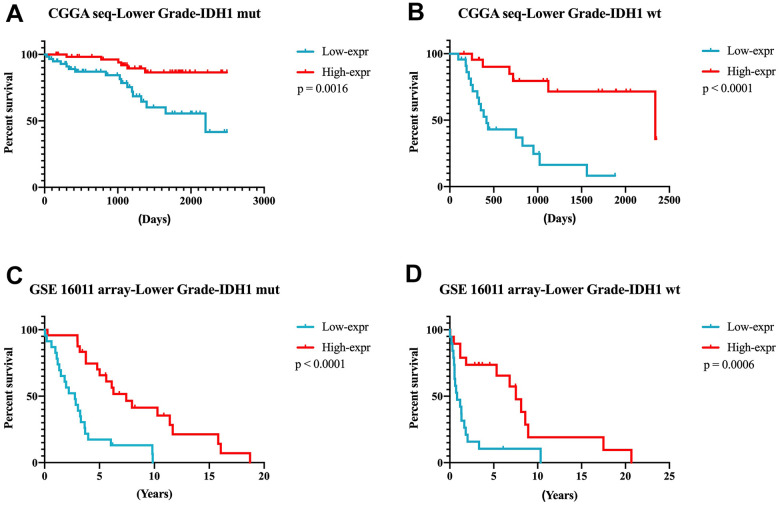
**DZIP3 expression could predict the survival time in *IDH1*-MUT and *IDH1*-WT subgroups in lower-grade gliomas.** (**A**, **B**) CGGA RNA-seq cohort; (**C**, **D**) GSE 16011 array cohort.

**Figure 6:**
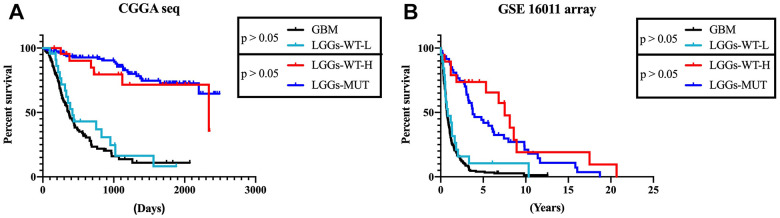
**DZIP3 could stratify lower-grade *IDH1*-WT gliomas patients into *IDH1*-MUT-like and GBM-like groups.** (**A**) CGGA RNA-seq cohort; (**B**) GSE 16011 array cohort.

According to the results mentioned earlier, higher *DZIP3* expression indicated a prolonged survival time in all grade and lower grade glioma, but not in GBM. Next, we focused on 356 lower-grade glioma patients for further research. In lower-grade glioma, *DZIP3* expression could predict the survival time in *IDH1* mutant and *IDH1* wild-type subgroups (Figure 5A–5D). *IDH1* mutation was a key genetic event that mainly occurs in lower grade gliomas, while the *IDH1* wild-type phenotype constituted the GBM group. *IDH1* wild-type lower-grade glioma was a group of patients with high heterogeneity. As shown in Figure 6A, *DZIP3* classified *IDH1* wild-type lower-grade glioma into GBM-like and *IDH1* mutation lower grade-like groups with similar survival tendencies. Also, these results could be validated in GSE 16011 array cohort (Figure 6B), indicating that the introduction of *DZIP3* could provide the basis for accurate diagnosis and treatment of glioma, especially lower-grade glioma.

## DISCUSSION

The prognosis of patients remains extremely poor, despite aggressive therapy [15]. WHO 2007 glioma classification mainly relies on the morphology and IHC evaluation [16]. Malignant gliomas can be divided into three grades: WHO grade II–IV. WHO grade II and III gliomas are usually described as lower-grade glioma, whereas GBM refers to WHO grade IV glioma [17]. According to 2016 WHO criteria, except for diffuse astrocytic and oligodendroglial tumors, the *IDH1* genotypes and status of chromosome arms *1p* and *19q* have been incorporated into the classification of gliomas [3, 17]. The glioma patients with tumors carrying a mutation in *IDH1* and chromosome *1p/19q* co-deletion could have a prolonged survival [18, 19]. The classification criteria published in 2016 were more precise than those in 2007. Ueki et al. indicated that *IDH*-mutated astrocytomas with 19q-loss constitute a subgroup with improved prognosis [20]. Yang et al. suggested that *IDH1* and *TERT* mutation in lower-grade glioma patients is responsible for prolonged survival time compared with other groups [21]. Additionally, the development of sequencing technology in various studies has focused on the stratification of glioma. Based on gene expression profiles, Wu et al. developed a novel molecular classification of *IDH*-mutant GBM with different clinical characteristics [11]. Moreover, in lower grade glioma, Cheng et al. established a five-gene signature to stratify *IDH1*-mutant lower-grade glioma with a distinct prognosis [9]. However, only a few studies have focused on *IDH1* wild-type lower-grade glioma patients.

**Figure 7:**
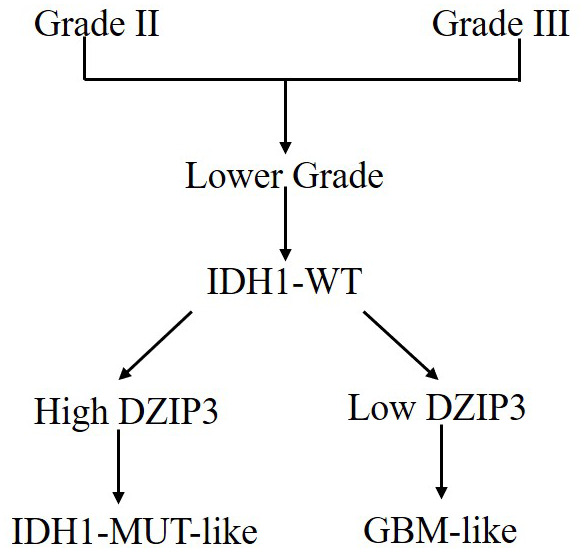
***IDH1*-MUT-like and GBM-like model for classification of lower-grade *IDH1*-WT gliomas based on DZIP3 expression.**

In the current study, we utilized two independent cohorts to deduce the role of *DZIP3* in glioma. DZIP3, also known as DAZ-interacting zinc finger protein 3, is an RNA-binding RING-type ubiquitin ligase and involved in various biological functions in glioma. Firstly, we tested the expression of *DZIP3* between different WHO grades and *IDH1* statuses and indicated that *DZIP3* expression is lower in GBM and *IDH1* wild-type groups. Moreover, based on *DZIP3* expression, we could reclassify the *IDH1* wild-type lower-grade glioma (Figure 7). Lower-grade glioma patients with lower *DZIP3* expression have similar survival time with GBM, while the survival time of the other patients is similar to that of the *IDH1* mutation group. In addition, we analyzed the *DZIP3* correlated genes using DAVID software. Biomedical analyses revealed that DZIP3 is significantly related to angiogenesis, which is the formation of new blood vessels and a prominent hallmark of glioma [22]. This neovascularization allows for increased oxygen and nutrients to be distributed to rapidly dividing tumor cells [23]. Recent studies designated CD31 (also known as PECAM) as the optimal marker of neoangiogenesis [24]. Herein, we verified the relationship between DZIP3 protein expression and CD31-MVD via IHC method, the results indicated that CD31-MVD was lower in higher DZIP3 protein expression samples.

Taken together, DZIP3 exerts a considerable effect on clinical and molecular conditions via the angiogenesis pathway. Thus, assessing *DZIP3* might aid in precise diagnosis of glioma, especially lower grade glioma, and provide a new target for glioma diagnosis and treatment. Therefore, using *DZIP3* target medicine for the treatment of glioma warrants for further investigation.

## MATERIALS AND METHODS

### Patients and samples

A total of 590 patients with WHO grade II–IV glioma (325 cases from the CGGA RNA-seq cohort and 265 cases from GSE 16011 array cohort) were included in this study. The CGGA RNA-seq cohort (http://www.cgga.org.cn) comprised of 109 grade II, 72 grade III, and 144 grade IV patients. Moreover, 24 grade II, 85 grade III, and 159 grade IV patients constituted the GSE 16011 array cohort (https://www.ncbi.nlm.nih.gov/geo/query/acc.cgi?acc=GSE16011). The histological diagnoses were determined according to the WHO 2007 criteria [25]. As described previously, the overall survival (OS) time was defined as the period from surgery to death [26]. The present study was approved by the Ethics Committee of Beijing Ditan Hospital and Beijing Tiantan Hospital.

### Immunohistochemistry for DZIP3 and CD31 expression

The expression of DZIP3 and CD31 proteins was assessed by IHC. The tumor tissues excised during the operation were immediately placed in 10% formalin for fixation, followed by dehydration, paraffin embedding, and sectioning. Anti-DZIP3 (Abcam) and CD31 (Zhong Shan, Beijing) antibodies were used at a dilution of 1:100. These results were confirmed by two neuropathologists individually. DZIP3 protein expression (semi-quantitative scoring) = expression intensity × expression area. Expression intensity was scored using a 4-point scale from 0 to 3. Expression area was scored using a 5-point scale from 0 to 4 [27]. Moreover, 0-6, 7-9 and 10-12 represented +, ++, +++ respectively.

### Counts of microvessel density (MVD)

MVD was based on CD31 immunoreactivity. We attained microscopy of CD31 at low magnification (×20) and three tumor areas with the highest density of distinctly highlighted microvessel (hot spot) within each section were selected for quantitation of MVD. Counts were performed on these fields in the hot spot at ×200 magnification [28].

### Bioinformatic analysis

Pearson’s correlation analysis explored the genes related to *DZIP3* through the R language. Negatively-related genes with correlation coefficient >0.4 were input into GO analysis in DAVID Bioinformatics Resources 6.8 to understand the biological functions of *DZIP3*.

### Statistical analysis

R language, GraphPad Prism 5, and SPSS 22.0 were used for statistical analyses. Student’s *t*-test was used to explore the diversity of *DZIP3* expression between different clinical and molecular groups. Kaplan–Meier analysis was performed to estimate the survival time of different subgroups, and a log-rank test assessed the prognostic differences. Univariate and multivariate Cox regression analyses were used to determine that DZIP3 is an independent factor to predict the survival time. *p* < 0.05 was considered as statistically significant [29].
